# Prevalence and intensity of avian malaria in a quail hybrid zone

**DOI:** 10.1002/ece3.7645

**Published:** 2021-05-19

**Authors:** Allison M. Roth, Carl N. Keiser, Judson B. Williams, Jennifer M. Gee

**Affiliations:** ^1^ Department of Biology University of Florida Gainesville FL USA; ^2^ Department of Ecology and Evolutionary Biology Princeton University Princeton NJ USA; ^3^ Present address: Department of Surgery Duke University Raleigh NC USA; ^4^ Present address: James San Jacinto Mountains Reserve University of California – Riverside University of California Natural Reserve System Idyllwild CA USA

**Keywords:** blood parasite, California quail, *Callipepla*, Gambel's quail, *Haemoproteus lophortyx*, species barriers

## Abstract

Hybrid zones have been described as natural laboratories by researchers who study speciation and the various mechanisms that may affect gene flow. The evolutionary consequences of hybridization depend not only on reproductive compatibility between sympatric species, but also on factors like vulnerability to each other's predators and parasites. We examined infection patterns of the blood parasite *Haemoproteus lophortyx*, a causative agent of avian malaria, at a site in the contact zone between California quail (*Callipepla californica*) and Gambel's quail (*C. gambelii*). Controlling for the potential influence of sex and year, we tested whether species identity predicted infection status and intensity. We found that infection prevalence was lower in California and hybrid quail compared with Gambel's quail. However, infected California and hybrid quail had higher infection intensities than Gambel's quail. California and hybrid quail exhibited no significant differences in prevalence or intensity of infection. These findings suggest that infection by *H. lophortyx* has the potential to influence species barrier dynamics in this system; however, more work is necessary to determine the exact evolutionary consequences of this blood parasite on hybridization.

## Graphical abstract

We examined infection patterns of the blood parasite *Haemoproteus lophortyx*, a causative agent of avian malaria, at a site in the contact zone between California quail (*Callipepla californica*) and Gambel's quail (*C. gambelii*). We found that California and hybrid quail had lower infection prevalence but higher infection intensities than Gambel's quail. Our results suggest that infection by *H. lophortyx* has the potential to influence species barrier dynamics in this system.

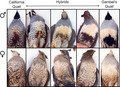

## 1 INTRODUCTION

Understanding the proximate and ultimate mechanisms underlying the maintenance of species barriers in localities where closely related congeners overlap is a major focus of evolutionary biology. Recently, parasitism has been put forth as a mechanism that may work to promote the formation, preservation, or breakdown of species barriers (reviewed in Theodosopoulos et al., 2019). Although tests of this hypothesis may be conducted in the laboratory (e.g., Brucker & Bordenstein, 2013; Goldberg et al., 2005; González et al., 2014; Hedrick et al., 2006; Liang et al., 2018), hybrid zones provide scientists with the opportunity to study the effects of factors influencing gene flow, such as parasitism, in a natural setting (Harrison & Larson, 2014; Kenney & Sweigart, 2016; Mořkovský et al., 2018; Theodosopoulos et al., 2019). Hybridization may have important ecological and/or evolutionary consequences for hybridizing host species as well as their associated parasites, and in order to better understand these consequences, it is important to examine coevolutionary dynamics between hosts and their parasites across hybrid zones (Hafner et al., 1998; Reullier et al., 2006; Theodosopoulos et al., 2019; Tompkins et al., 2003).

For parasites, host hybrid zones can function as population sinks, population sources, or as “bridges” that enable the colonization of a new host species (Floate & Whitham, 1993; Strauss, 1994; Whitham, 1989). Parasite specificity may limit or facilitate a parasite's range expansion into a new host species, and parasites with lower specificity are predicted to move more easily between host species (Bensch et al., 2000; Reullier et al., 2006; Ricklefs & Fallon, 2002; Ricklefs et al., 2004). Nevertheless, because hybrids may have a range of phenotypes intermediate to that of their parental species, hybrid zones may facilitate host shifting by parasites from one parental species to the other, via hybrid individuals, even in cases where host specificity is relatively high (Floate & Whitham, 1993).

For hosts, parasites may alter the direction and magnitude of gene exchange between two hybridizing species, and differential parasitism between hybrids and their parental species has the potential to reinforce or degrade host species barriers (e.g., Derothe et al., 2001; Parris, 2004; Goldberg et al., 2005; Hedrick et al., 2006; Brucker & Bordenstein, 2013; González et al., 2014; Guttel & Ben‐Ami, 2014; Maynard et al., 2016; Eastwood et al., 2017; Liang et al., 2018; reviewed in Theodosopoulos et al., 2019). If hybrids experience an overall reduction in fitness, due to higher susceptibility, higher exposure, and/or lower tolerance to parasites than parental species, backcrossing, resulting in introgression of parental genes, should be minimized, leading to the reinforcement of species barriers (Baird & de Bellocq, 2019; Grant & Grant, 2008; Moulia, 1999; Theodosopoulos et al., 2019). Alternatively, in instances where no other factors work to maintain species barriers, these barriers may be eroded if hybrids have intermediate or greater fitness than parental species, due to reduced susceptibility, reduced exposure, and/or increased tolerance to parasitism (Baird & de Bellocq, 2019; Theodosopoulos et al., 2019). Past work has provided support for both parasitic driven maintenance (e.g., Brucker & Bordenstein, 2013; Derothe et al., 2001; Goldberg et al., 2005; González et al., 2014; Parris, 2004) and erosion (e.g., Eastwood et al., 2017; Guttel & Ben‐Ami, 2014; Hedrick et al., 2006; Liang et al., 2018; Maynard et al., 2016) of species barriers, across a range of animal taxa, with ca. 37% of studies examined in a 2019 review suggesting that hybrids are more negatively affected by parasites, and ca. 41% of studies suggesting that hybrids are less negatively affected by parasites, compared with parental species (Theodosopoulos et al., 2019). Nevertheless, it is important to bear in mind that, although differences in parasite load between parental and hybrid hosts are often used as a proxy for relative hybrid fitness (e.g., Theodosopoulos et al., 2019), differences between species in tolerance may distort the link between parasite load and fitness (Baird & de Bellocq, 2019). For example, transgressive segregation may lead to extreme tolerance in hybrids compared with their parental species (Baird & de Bellocq, 2019). Hybrids could therefore have high parasite loads while retaining relatively high fitness (Baird & de Bellocq, 2019).

Differences in parasite susceptibility or fitness costs may arise between hybrids and their parental species due to immunological factors (Baack & Rieseberg, 2007; Baird & de Bellocq, 2019; Grossen et al., 2014; Guttel & Ben‐Ami, 2014; Nadachowska‐Brzyska et al., 2012; Theodosopoulos et al., 2019; Zhang et al., 2017). Hybrids may be better or worse equipped than parental species to fight off infection or may be more or less tolerant to high parasite loads. Indeed, hybrid vigor may exist with respect to parasite resistance or tolerance, due to the admixture of locally adapted alleles conferring resistance or tolerance from both parental species, the generation of transgressive phenotypes for resistance or tolerance (i.e., hybrids may possess phenotypes that are extreme compared with parental phenotypes), interactions between hybrid immune systems and other transgressive traits, such as body size, and/or higher MHC diversity (Rieseberg et al., 1999; Baack & Rieseberg, 2007; Nadachowska‐Brzyska et al., 2012; Grossen et al., 2014; Guttel & Ben‐Ami, 2014; Zhang et al., 2017; Theodosopoulos et al., 2019; Baird & de Bellocq, 2019; although it is important to note that increased MHC diversity does not always result in heightened immune response; Sommer, 2005; Sommer et al., 2014). Conversely, hybrids may have decreased resistance or tolerance, compared with parental species, or it may be more costly for hybrids to mount an immune response, due to a higher stress response, fewer resources, and/or because genetic mixing may lead to debilitated immune function and/or metabolic processes (Dupont & Crivelli, 1988; Moulia, 1999; Theodosopoulos et al., 2019). Hybrids may also exhibit intermediate resistance or tolerance compared with parental species if they possess intermediate MHC diversity or inherit a combination of alleles related to resistance or tolerance (Theodosopoulos et al., 2019).

In addition to differences in host–parasite interactions arising from immunological factors, hybrids may exhibit behaviors or have an ecology that is distinct from their parental types, such as different intra‐ and interspecific social relationships, food resources, and habitat use, which could lead to higher or lower parasite transmission or infection intensity in hybrids, compared with parental species (Hiadlovská et al., 2013; Theodosopoulos et al., 2019). For example, hybrid *Mus musculus musculus*; Linnaeus, 1758 × *Mus musculus domesticus*; Schwarz and Schwarz, 1943 house mice have been demonstrated to show transgressive behavioral phenotypes, with hybrids taking longer to enter an experimental arena, compared with either parental species (Hiadlovská et al., 2013). This increased latency could have implications for behavior under natural conditions and may lead to differential parasite exposure between hybrid and parental species. Interestingly, various other studies in this system have indeed demonstrated differences in parasitism between hybrid and parental mice (e.g., Sage et al., 1986; Moulia et al., 1991; Moulia et al., 1995; Derothe et al., 2001; Derothe et al., 2004; but see Derothe et al., 1999), although potential underlying behavioral mechanisms were not examined. Furthermore, environmental conditions may modulate differences in parasitism between hybrid and parental species (Theodosopoulos et al., 2019). For example, the likelihood of *Haemoproteus* infection appears to increase with increasing elevation for myrtle warblers, *Setophaga coronata*; Linnaeus, 1766 and decrease with increasing elevation for Audubon's warblers, *Setophaga auduboni*; Townsend, 1837 and for myrtle warbler × Audubon's warbler hybrids (Cozzarolo et al., 2018).

California quail, *Callipepla californica*; Shaw, 1798 and Gambel's quail, *Callipepla gambelii*; Gambel, 1843 are sister species of medium‐sized (ca. 150–200 g), highly social, sexually dichromatic, nonmigratory New World quail (Odontophoridae; Gee, 2003, 2004; Hosner et al., 2015; Leopold, 1977; Zink & Blackwell, 1998). California quail are native to the western United States and Baja California, preferring chaparral and semiarid scrub, while the natural ranges of Gambel's quail span the Mojave and Sonoran Deserts, with these quail preferring more arid environments (Leopold, 1977). California and Gambel's quail hybridize readily under captive and natural conditions, giving rise to a hybrid zone, which straddles a narrow ecotone (roughly 20–30 km), where their ranges overlap (Gee, 2003, 2004; Johnsgard, 1971). Nevertheless, it is currently unclear whether parasitism plays a role in mediating species barrier dynamics in this system.

Using 4 years of data, we explored infection patterns of *Haemoproteus lophortyx*, a parasite which spends part of its lifecycle infecting the red blood cells of its quail hosts, at a site in the northern region of the contact zone between California quail and Gambel's quail. Our aim was to assess whether *H. lophortyx* has the potential to influence species barriers in this host system. Controlling for the potential influence of sex and year, we compared the status and intensity of *H. lophortyx* infection in California quail, Gambel's quail, and their hybrids.

## 2 METHODS

### 2.1 Study System and field methods

The California quail × Gambel's quail hybrid zone is located along an ecological transition between the relatively mesic habitat of California quail to the relatively xeric habitat of Gambel's quail. In the hybrid zone, California and Gambel's quail can live in stable mixed‐species coveys during the nonbreeding season, and individuals disband for breeding in the spring (Gee, 2003, 2004; Leopold, 1977; Zonana et al., 2019, 2021). There is no evidence for assortative mating in the hybrid zone, and local gene exchange occurs frequently in disjunct patches of species overlap (Gee, 2003, 2004). Hybrids of all classes are present in the hybrid zone and hybrids can easily comprise at least 20% of the population, depending upon the ecological conditions (Gee, 2004).

The genus *Haemoproteus* is a diverse group of parasitic alveolates that parasitize a range of avian species (e.g., Ayadi et al., 2018; Iezhova et al., 2011; Levin et al., 2012; Ricklefs et al., 2005; Swanson et al., 2014; Valkiūnas et al., 2007, 2010, 2013). Along with parasites of the closely related genus *Plasmodium*, *Haemoproteus* can cause the disease known as avian malaria (Asghar et al., 2015; Cosgrove et al., 2006; Olias et al., 2011; Richard et al., 2002). Furthermore, *Leucocytozoon*, a sister taxon to *Plasmodium* and *Haemoproteus*, has been shown to cause a disease known as leucocytozoonosis, which resembles avian malaria (Adler & McCreadie, 2019; Cosgrove et al., 2006). Unlike *Plasmodium*, *Haemoproteus* lineages appear to have high host specificity (Atkinson & Van Riper, 1991a; Ayadi et al., 2018; Bensch et al., 2000; Clark & Clegg, 2017; Loiseau et al., 2017). *Haemoproteus lophortyx*; O'Roke, 1929 may cause anemia, prostration, and death in various quail species, including California quail, Gambel's quail, and bobwhite quail, *Colinus virginianus*; Linnaeus, 1758 (Cardona et al., 2002; Gullion, 1957; Herman & Glading, 1942; Mullens et al., 2006; O’Roke, 1928, 1930, 1932; Samour, 2016; Tarshis, 1955, 1958). There exists no evidence for sex differences in infection in California quail (Herman & Glading, 1942); however, no studies have examined whether sex differences in infection may exist in Gambel's or hybrid quail. Past work has demonstrated that *H. lophortyx* may be spread by several vectors including hippoboscid flies (*Lynchia hirsuta*; Ferris 1927 and *Stilbometopa impressa*; Bigot, 1885; both of which are obligate ectoparasites) and biting midges (*Culicoides* spp—especially *C. bottimeri*; Wirth, 1955; Tarshis, 1955, 1958; Mullens et al., 2006; Samour, 2016).

**FIGURE 1:**
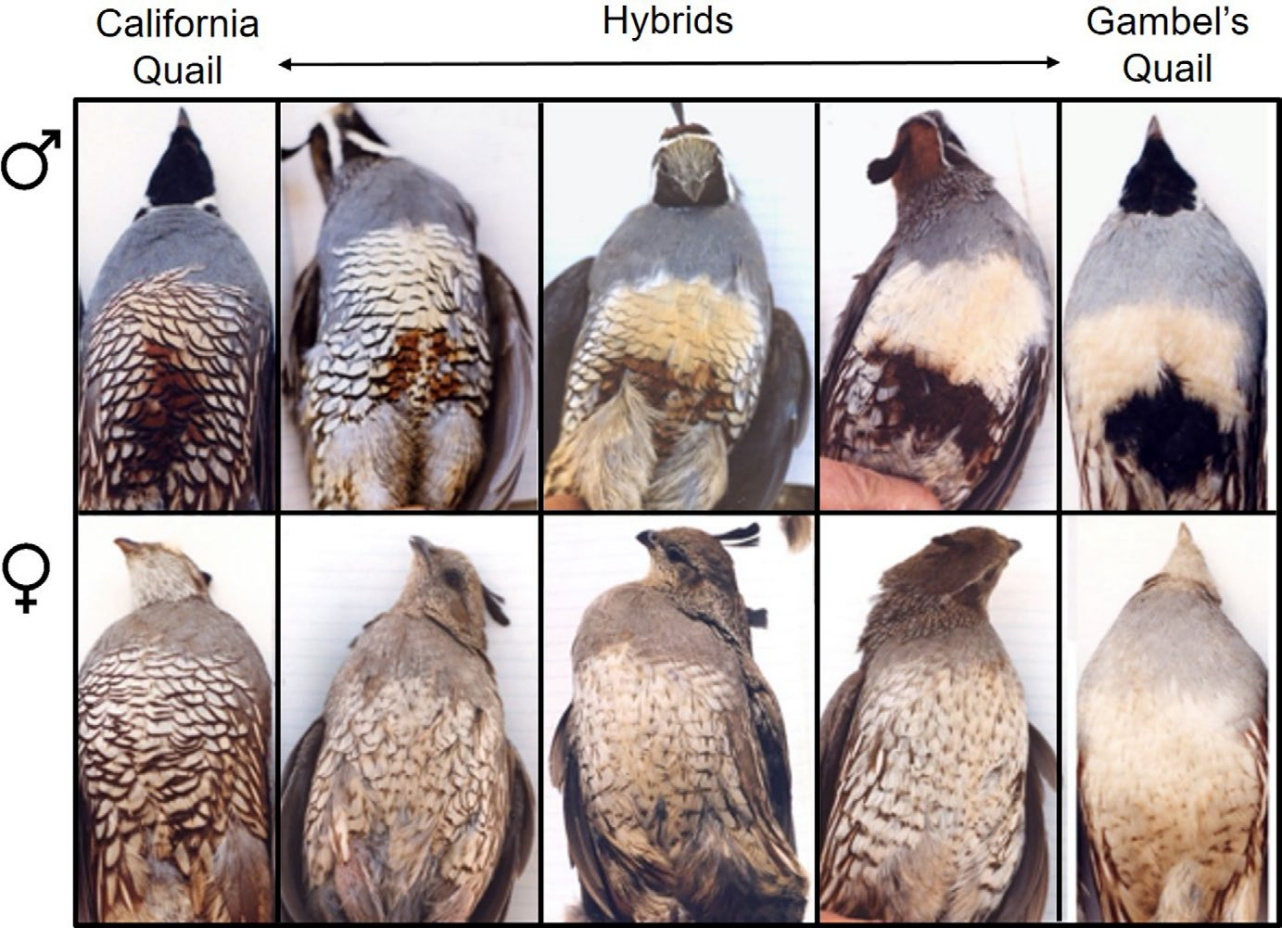
The spectrum of morphological traits we examined when characterizing male (top row) and female (bottom row) birds as California quail (leftmost vertical panel), hybrid quail (3 middle vertical panels), or Gambel's quail (rightmost vertical panel). Moving from California quail to Gambel's quail, the following patterns are observed: scaled breast, brown abdominal patch, overall blue body, chestnut colored cap and flanks, gray forehead, and shorter plume versus the buffy unscaled breast, black belly patch, overall tan body, rust colored cap and flanks, blackish forehead, and longer plume. Photographs by JMG

We trapped quail using seed‐baited, walk‐in funnel traps between January and September from 1998 to 2001 at a site called Royal Carrizo (33.6410°N, 116.4253°W; Figure S1) in Southern California, which consists of pinyon–juniper woodland habitat at ca. 3,000’. Please see Zonana et al. (2019), Zonana et al. (2021) for detailed maps of our study site in relation to each species' range. Hybrid reproductive success at this sympatric study site is moderate, based on comparisons of clutch size and hatching success with the two parental species (Gee, 2003). We assigned birds as California quail, Gambel's quail, or hybrids, based on morphological features, which are tightly correlated with genotype (Figure 1; Gee, 2004). Although we did not collect data on the age (plumage characteristics can only be used to assess whether an individual is younger or older than 1 year; Roth, Gee, et al., 2021; Roth, Keiser, et al., 2021; Williams, 1959) or the weight of all of the captured individuals, we sexed each bird (Figure 1). We banded birds for individual identification and collected blood samples to test for *H. lophortyx* infection. We collected blood from the left brachial vein into a microcapillary tube. We smeared the blood onto a glass slide, which we air‐dried and fixed in 95% ethanol before Giemsa staining. This work was conducted under California State Fish and Game permit SC 949 and was approved by Princeton University's Institutional Animal Care and Use Committee.

### 2.2 Parasite quantification

Criteria for *H. lophortyx* identification were based on the morphological descriptions given by O’Roke (1928) and Atkinson and Van Riper (1991b). Using an oil immersion lens at 100× magnification, we scanned each blood smear for 15 min, in line with methods from previous work (e.g., Durrant et al., 2006; Hille et al., 2007; Salmani et al., 2011; Van Riper et al. 1986), which resulted in a minimum of 30 fields of view being examined. We obscured all information about the sample before parasite quantification. We counted the number of *H. lophortyx* infected erythrocytes seen within these 15 min. We also counted the total number of erythrocytes present in a single field of view and then multiplied this number by the number of fields of view examined, to estimate the total number of erythrocytes scanned. At 100× magnification, a mean ± *SD* of 184 ± 73 erythrocytes per field of view per sample was examined.

There exists a fair amount of variation in how researchers conduct parasite quantification using blood smears (e.g., Dadam et al., 2019; Davis et al., 2013; Durrant et al., 2006; Gutiérrez‐López et al., 2016; Hille et al., 2007; Kelly et al., 2016; Rätti et al., 1993; Salmani et al., 2011; Shurulinkov et al., 2018; Staats & Schall, 1996; Van Riper et al. 1986). Thus, in order to check the robustness of our results, we reran the analyses described below using a subset of the data where at least 10,000 erythrocytes were examined for each sample, in line with methods from other studies (Kelly et al., 2016; Rätti et al., 1993; Shurulinkov et al., 2018; Staats & Schall, 1996). Of the 209 blood smears in which a minimum of 30 fields of view were scanned over the course of 15 min, there were 189 samples in which 10,000 or more erythrocytes were examined. Sixty‐three of these showed evidence of *H. lophortyx* infection.

### 2.3 Statistical methods

We used R 3.5.2 (R Core Team, 2018) for all analyses and, controlling for the potential influence of sex and year, we explored whether species could predict the status and intensity (i.e., proportion of infected erythrocytes) of *H. lophortyx* infection. To examine infection status, we used the glmer function in the “lmerTest” package (Kuznetsova et al., 2017) to run a generalized linear mixed model with a binomial error distribution and the status of infection (1 = infected, 0 = uninfected) as the response. To examine infection intensity, we ran a similar model, but instead, included the ratio of infected to uninfected erythrocytes as the response. For this analysis, we only examined the subset of individuals that were infected. For both analyses, we included species (i.e., California quail, Gambel's quail, or hybrids), sex, and year as fixed effects, and individual identity and month as random effects. We included month as a random effect given that past work has demonstrated seasonal fluctuations in *H. lophortyx* infection in quail (Cardona et al., 2002; Tarshis, 1955). Because species was a categorical variable, we examined whether the overall effect of species was significant by comparing models with and without species, using likelihood ratio tests. Similarly, given that year and sex were also categorical, we used likelihood ratio tests to compare models with and without year, and to compare models with and without sex, to determine the overall effect of these potentially influential variables.

## 3 RESULTS

We tested for the presence of *H. lophortyx* in 193 quail (72 California quail, 27 Gambel's quail, and 94 hybrids), 13 of which were sampled twice across years (3 California quail, 3 Gambel's quail, and 7 hybrids), and one California quail which was sampled three times, for a total of 208 blood smears across 4 years (1998: *N* = 61 blood smears; 1999: *N* = 32 blood smears; 2000: *N* = 102 blood smears; 2001: *N* = 13 blood smears; see Table S1). Of the 208 blood smears examined, 69 (~33%) showed signs of *H. lophortyx* infection (24 California quail smears (~31%), 16 Gambel's quail smears (~53%), and 29 hybrid smears (~29%)), and infection intensity ranged from 4.700e−5–0.013 *H. lophortyx*/cell with a mean ± *SD* of 0.002 ± 0.002 *H. lophortyx*/cell (California quail: range = 5.710e−5–0.013 *H. lophortyx*/cell, mean ± *SD* = 0.003 ± 0.003 *H. lophortyx*/cell; Gambel's quail: range = 6.000e−5–0.001 *H. lophortyx*/cell, mean ± *SD* = 3.686e−4 ± 2.689e−4 *H. lophortyx*/cell; hybrid quail: range = 4.700e−5–0.009 *H. lophortyx*/cell, mean ± *SD* = 0.001 ± 0.002 *H. lophortyx*/cell).

**FIGURE 2:**
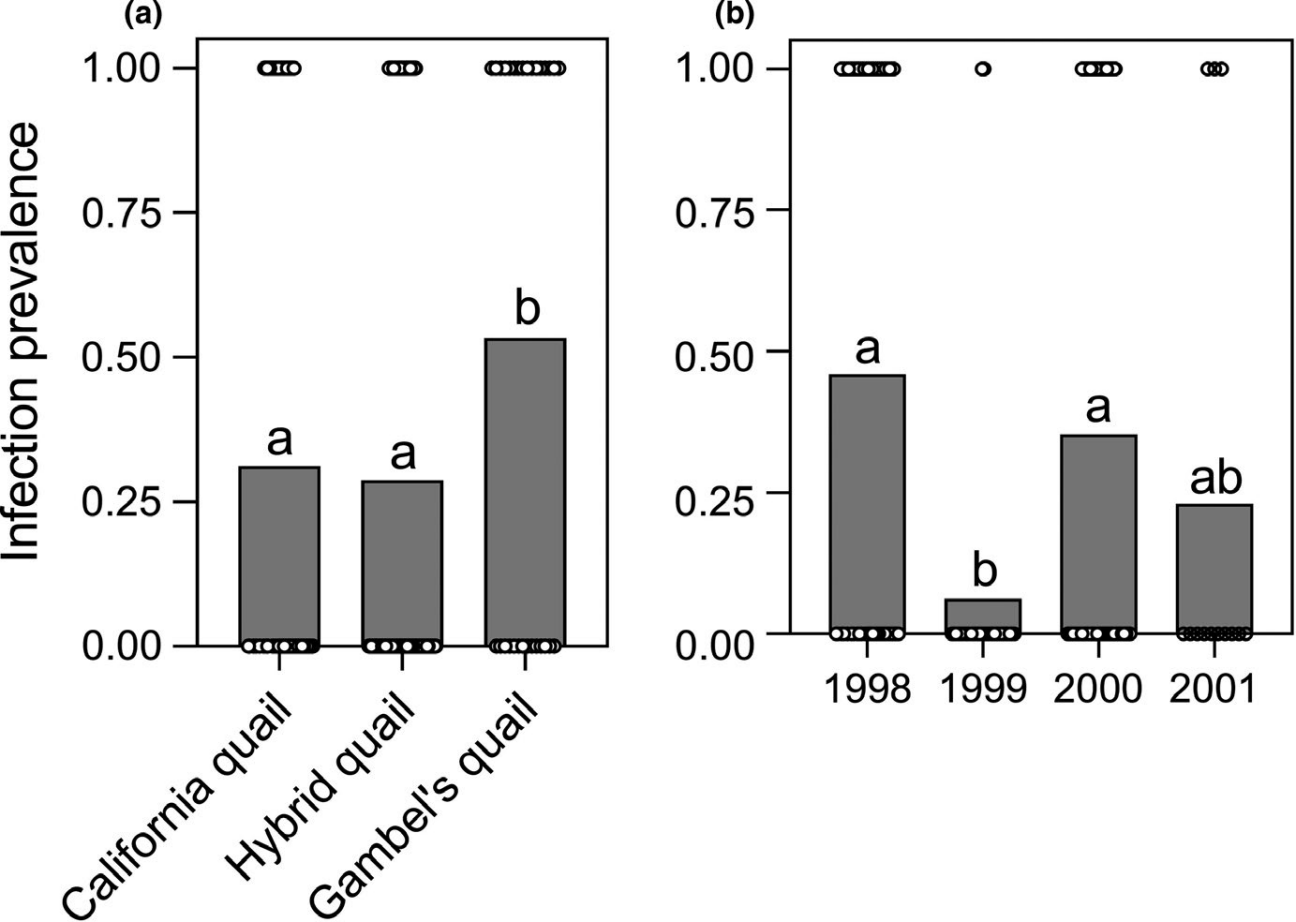
Lowercase letters denote statistical differences. (a) Infection status of *Haemoproteus lophortyx* in California quail, hybrid quail, and Gambel's quail when infection was determined by scanning blood smears for 15 min at 100× magnification. (b) Infection status of *Haemoproteus lophortyx* in 1998, 1999, 2000, and 2001 when infection was determined by scanning blood smears for 15 min at 100× magnification

**FIGURE 3:**
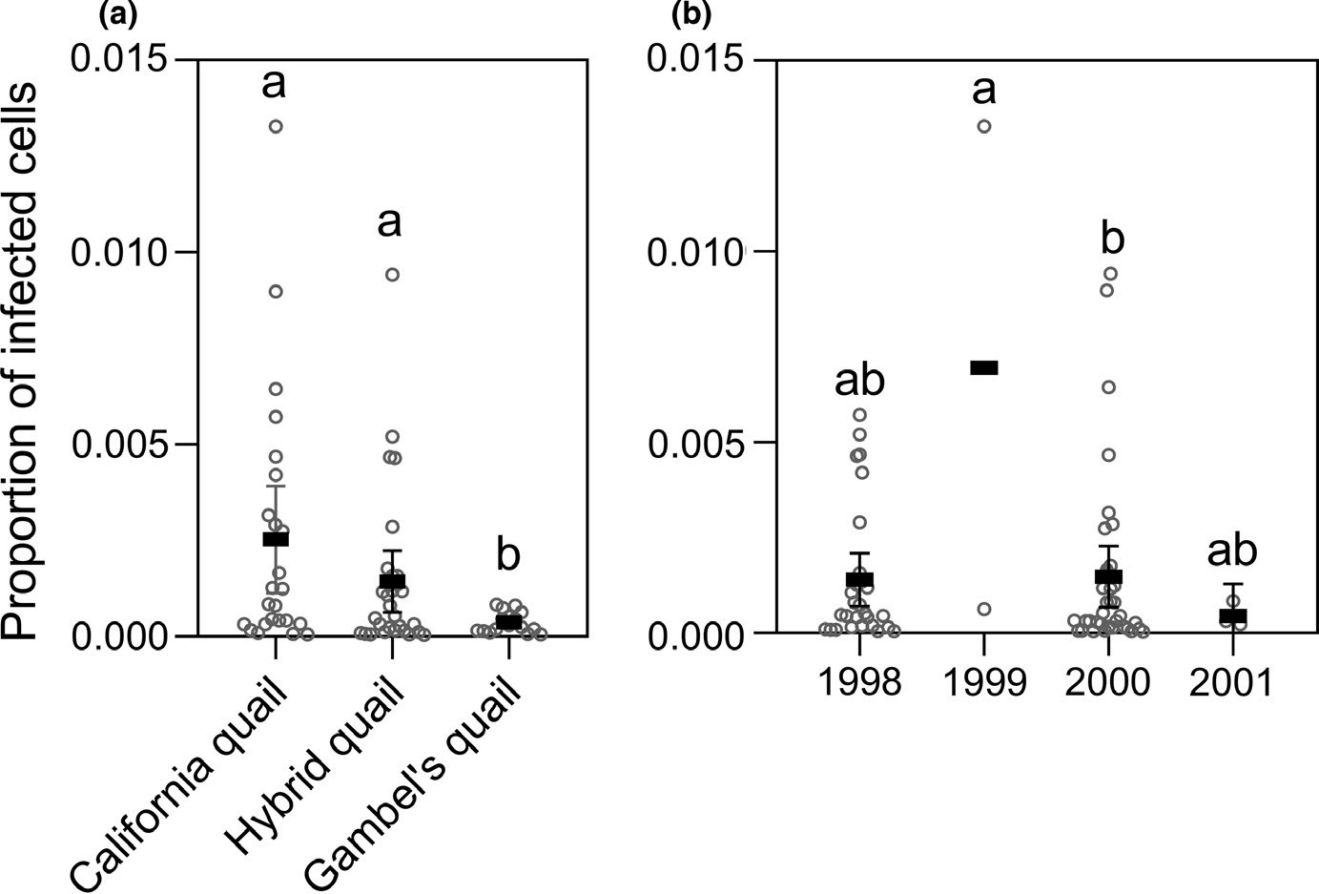
Lowercase letters denote statistical differences. (a) Infection intensities of *Haemoproteus lophortyx* in California quail, hybrid quail, and Gambel's quail when infection was determined by scanning blood smears for 15 min at 100× magnification. (b) Infection intensities of *Haemoproteus lophortyx* in 1998, 1999, 2000, and 2001 when infection was determined by scanning blood smears for 15 min at 100× magnification

For the analysis examining infection status, we found that significantly more Gambel's quail were infected than either California or hybrid quail, but we found no significant difference between hybrids and California quail (i.e., Gambel's quail > hybrid quail ≈ California quail; Table 1; Figure 2a). In contrast, for the analysis examining the intensity of infection in the subset of individuals that were infected, California and hybrid quail had significantly higher proportions of *H. lophortyx*/cell than Gambel's quail (Table 2; Figure 3a). Again, there was no significant difference between hybrids and California quail (i.e., Gambel's quail < hybrid quail ≈ California quail, Table 2; Figure 3a). There was a significant overall effect of species on *H. lophortyx* infection status (chi‐squared = 8.997, *df* = 2, *p* = .011) and intensity (chi‐squared = 8.162, *df* = 2, *p* = .017). There was also a significant overall effect of year on infection status (chi‐squared = 21.560, *df* = 3, *p* < .001), with significantly fewer individuals infected in 1999 than in 1998 or 2000 (Table S2; Figure 2b). Furthermore, there was a significant overall effect of year on infection intensity (chi‐squared = 17.367, *df* = 3, *p* = .001), with significantly higher intensities of infection in 1999 compared with 2000 (Table S3; Figure 3b). It is, however, important to note that we had a limited sample size for both 1999 and 2001, for this analysis, as we only examined the subset of individuals that were infected. Given this, these results should be taken with caution. We found no significant overall effect of sex on infection status (chi‐squared = 0.225, *df* = 1, *p* = .635) or intensity (chi‐squared = 0.491, *df* = 1, *p* = .484).

**TABLE 1:** Output of a generalized linear mixed model with a binomial error distribution showing the effects of species (i.e., California quail, Gambel's quail, California × Gambel's quail hybrid), year (1998, 1999, 2000, 2001), and sex on *Haemoproteus lophortyx* infection status, when infection was determined by scanning blood smears for 15 min at 100× magnification (*N* = 208)

Predictor	Est ± *SE*	*Z*	*p*
Reference Class: California Quail			
Species: Hybrid Quail	−0.012 ± 0.355	−0.033	.973
Species: Gambel's Quail	1.394 ± 0.519	2.685	.**007**
Year: 1999	−2.734 ± 0.817	−3.346	.**001**
Year: 2000	−0.098 ± 0.374	−0.261	.794
Year: 2001	−0.972 ± 0.764	−1.272	.204
Sex: Male	0.173 ± 0.341	0.507	.612
Reference Class: Hybrid Quail			
Species: California Quail	0.012 ± 0.355	0.033	.973
Species: Gambel's Quail	1.406 ± 0.504	2.788	.**005**
Year: 1999	−2.734 ± 0.817	−3.346	.**001**
Year: 2000	−0.098 ± 0.374	−0.261	.794
Year: 2001	−0.972 ± 0.764	−1.272	.204
Sex: Male	0.173 ± 0.341	0.507	.612
Reference Class: Gambel's Quail			
Species: California Quail	−1.394 ± 0.519	−2.685	.**007**
Species: Hybrid Quail	−1.406 ± 0.504	−2.788	.**005**
Year: 1999	−2.734 ± 0.817	−3.346	.**001**
Year: 2000	−0.098 ± 0.374	−0.261	.794
Year: 2001	−0.972 ± 0.764	−1.272	.204
Sex: Male	0.173 ± 0.341	0.507	.612

Note

Bold valuese indicates *p* < .05.

Individual identity and month were included as random effects.

The results presented in each subsection of this table represent the same model with different species coded as the reference class. Presenting the same model with each species coded as the reference class allows for a comparison of *H. lophortyx* infection status between each pair of species. For year, the reference class is 1998 (see Table S2 for a comparison between years). For sex, the reference class is female.

**TABLE 2:** Output of a generalized linear mixed model with a binomial error distribution showing the effects of species (i.e., California quail, Gambel's quail, California × Gambel's quail hybrid), year (1998, 1999, 2000, 2001), and sex on the intensity of *Haemoproteus lophortyx* infection (i.e., proportion of infected erythrocytes), when infection was determined by scanning blood smears for 15 min at 100× magnification (*N* = 69)

Predictor	Est ± *SE*	*Z*	*p*
Reference Class: California Quail			
Species: Hybrid Quail	−0.474 ± 0.374	−1.269	0.204
Species: Gambel's Quail	−1.416 ± 0.486	−2.913	**0.004**
Year: 1999	0.319 ± 0.305	1.045	0.296
Year: 2000	−0.088 ± 0.290	−0.304	0.761
Year: 2001	−0.770 ± 0.827	−0.932	0.351
Sex: Male	−0.244 ± 0.356	−0.686	0.493
Reference Class: Hybrid Quail			
Species: California Quail	0.474 ± 0.374	1.269	0.204
Species: Gambel's Quail	−0.942 ± 0.469	−2.010	**0.044**
Year: 1999	0.319 ± 0.305	1.045	0.296
Year: 2000	−0.088 ± 0.290	−0.304	0.761
Year: 2001	−0.770 ± 0.827	−0.932	0.351
Sex: Male	−0.244 ± 0.356	−0.686	0.493
Reference Class: Gambel's Quail			
Species: California Quail	1.416 ± 0.486	2.913	**0.004**
Species: Hybrid Quail	0.942 ± 0.469	2.010	**0.044**
Year: 1999	0.319 ± 0.305	1.045	0.296
Year: 2000	−0.088 ± 0.290	−0.304	0.761
Year: 2001	−0.770 ± 0.827	−0.932	0.351
Sex: Male	−0.244 ± 0.356	−0.686	0.493

Note

Bold valuese indicates *p* < .05.

Individual identity and month were included as random effects.

The results presented in each subsection of this table represent the same model with different species coded as the reference class. Presenting the same model with each species coded as the reference class allows for a comparison of *H. lophortyx* infection intensity between each pair of species. For year, the reference class is 1998 (see Table S3 for a comparison between years). For sex, the reference class is female.

We obtained qualitatively similar results when we examined a subset of the data where 10,000 or more erythrocytes were scanned (Appendix S1; Tables S4 and S5), suggesting that our findings were robust to different sampling methods.

## 4 DISCUSSION

The hybridization of two host species can have complex effects on host–parasite interactions. We found that, while there was no significant difference between hybrid California × Gambel's quail and California quail in *Haemoproteus lophortyx* infection status and intensity, both species had lower infection prevalence, but higher infection intensity, than Gambel's quail. Higher infection intensities may lead to a reduction in fitness due to increased costs of parasitism (e.g., increased energetic costs arising from direct resource competition with parasites, mounting an immune response, parasite removal, or repairing tissue damage; Delahay et al., 1995; Demas et al., 1997; Giorgi et al., 2001; Kyriazakis et al., 1998; Lutermann et al., 2013; Svensson et al., 1998). Low‐intensity chronic infections could, however, also have negative impacts on fitness (e.g., Puente et al., 2010; Asghar et al., 2011; Asghar et al., 2015; but see Ortego et al., 2008). Overall, our results suggest that, in this Galliform host system, infection by the blood parasite *H. lophortyx* has the potential to impact species barrier dynamics. Nevertheless, future work quantifying the relative fitness costs of *H. lophortyx* is needed to explore this possibility further.

We found that there were fewer *H. lophortyx* infected California and hybrid quail than Gambel's quail, but upon *H. lophortyx* infection, California and hybrid quail had higher infection intensities compared with Gambel's quail. This may appear counterintuitive, as one might expect relationships involving infection status and intensity to be similar in directionality. For example, a study conducted on three species of wild doves found that species which had higher likelihoods of *Haemoproteus columbae* infection had higher, rather than lower, infection intensities (Adriano & Cordeiro, 2001). There are two possible scenarios that may lead to the patterns observed in our study (see Table 3 for a complete summary of these scenarios and their potential implications).

**TABLE 3:** Summary of the two mutually exclusive explanations for our observed results and how each scenario is expected to affect species barrier dynamics, as well as the potential range expansion of *Haemoproteus lophortyx*

Scenario	Species		Species Barrier Maintenance or Breakdown	Hybrid zone acts as a bridge for *H. lophortyx* to expand its range?
	Gambel's Quail	California/Hybrid Quail		
1	*Host behavior/ecology or vector preference leads to higher exposure to *H. lophortyx*, which leads to higher infection prevalence *Have higher resistance to *H. lophortyx*, due to longer coevolution with the parasite, which leads to lower intensities of infection (i.e., they are better at fighting off infection once infected)	*Host behavior/ecology or vector preference leads to lower exposure to *H. lophortyx*, which leads to lower infection prevalence *Have lower resistance to *H. lophortyx*, due to shorter coevolution with the parasite, which leads to higher intensities of infection (i.e., they are worse at fighting off infection once infected)	Species barrier maintenance is expected if the higher intensity of infection in hybrid quail leads them to have lower fitness, compared with Gambel's quail	If Gambel's quail have a longer coevolution with *H. lophortyx* than California quail, hybrid zones may act as a bridge for the parasite to colonize California quail
2	*Have similar rates of exposure to *H. lophortyx* as California/hybrid quail, but have lower resistance, and are therefore more likely to become infected and are less able to completely clear infections, once infected *This leads to a high prevalence of low‐intensity, chronic infections	*Have similar rates of exposure to *H. lophortyx* as Gambel's quail, but have higher resistance, and are therefore better at resisting initial infection and clearing an infection, once infected *This leads to a low prevalence of high‐intensity infections, given that infection intensities are highest during the initial stages of infection	Species barrier breakdown is expected if chronic infection in Gambel's quail leads them to have lower fitness, compared with hybrid quail	n/a because the behavior/ecology of each quail species leads to similar exposure rates across species

First, it is possible that vector preference or host behavior/ecology causes Gambel's quail to experience higher rates of *H. lophortyx* exposure, compared with California quail, leading to higher infection prevalence in the former. For example, vectors may exhibit a host preference for Gambel's quail over California quail. Furthermore, the frequency, type, network structure, or duration of social interactions in Gambel's quail may be more amenable to the direct horizontal transmission of *H. lophortyx* carrying hippoboscid flies, compared with the social interactions of California quail. There is, for instance, some evidence to suggest that California quail may begin sampling potential mates during the nonbreeding season, which may influence social network dynamics in such a way that individuals spend disproportionately more time with preferred members of the opposite sex, compared with other members of the covey (Roth, Gee, et al., 2021; Roth, Keiser, et al., 2021). It is unclear whether Gambel's quail exhibit similar patterns, but if Gambel's quail distribute associations more evenly across covey mates during the nonbreeding season than California quail, they may be more likely to encounter an individual harboring a *H. lophortyx* infected hippoboscid fly and may thus be more likely to become infected. Although past work has found no evidence that quail at our study site are more likely to associate with individuals with similar species‐specific plumage or similar genetic ancestry, associations were inferred from spatiotemporal data (Zonana et al., 2021, 2019), and more work is needed to elucidate whether behavioral differences between species might influence the direct horizontal transmission of hippoboscid vectors.

Biting midges have been implicated in the transmission of *H. lophortyx* in bobwhite quail and may also transmit *H. lophortyx* in the quail species examined in our study (Mullens et al., 2006). It is possible that Gambel's quail are more likely to encounter biting midges compared with California quail due to differences in habitat use or activity patterns. Biting midges typically fare best in habitats with a high moisture content and an abundance of decaying material and may rest in high grasses, shrubs, trees, or animal shelters (Carpenter et al., 2008; Isberg, 2014; Kettle, 1977; Thompson et al., 2013). Interestingly, however, Gambel's quail tolerate aridity better than California quail and, in areas of sympatry, Gambel's quail are more likely to settle along washes than California quail (Calkins et al., 2020; Gee et al., 2020). It therefore seems unlikely that the habitat preferences of Gambel's quail would promote higher contact rates with biting midges, compared with California quail. Even so, female midges typically feed at dawn and/or dusk (Kettle, 1977), and if Gambel's quail are more active than California quail during these times, such activity patterns could lead Gambel's quail to encounter biting midges more frequently.

If Gambel's quail experience higher rates of *H. lophortyx* exposure, compared with California quail, due to their behavior/ecology, this may indicate a longer coevolutionary history between Gambel's quail and *H. lophortyx* than between California quail and *H. lophortyx*, leading Gambel's quail to have evolved higher resistance to *H. lophortyx* infection (i.e., they are better able to fight off infection). This would explain the relatively low infection intensities seen in Gambel's quail in our study. Moreover, if Gambel's quail have a longer coevolutionary history with *H. lophortyx* than California quail, it is possible that the hybrid zone may have acted as a bridge for *H. lophortyx* to expand its range from Gambel's quail to California quail.

Hybrid quail may have inherited a behavior/ecology which is similar to parental California quail, making them less likely to acquire the parasite than Gambel's quail. Furthermore, hybrid quail may not have inherited resistance alleles from parental Gambel's quail. If Gambel's quail have lower infection intensities than California and hybrid quail, due to higher resistance to infection, and if hybrid quail have reduced fitness compared with Gambel's quail, as a result of higher infection intensities, we would expect species barriers to be maintained.

A second scenario that would explain the patterns observed in our study hinges on the possibility that Gambel's quail may actually have *lower* resistance to *H. lophortyx* than California quail, causing them to exhibit a higher prevalence, but lower intensity of infection, compared with California quail. In this scenario, Gambel's quail may experience similar rates of exposure to *H. lophortyx* as California quail; however, Gambel's quail may be more likely to become infected and less likely to completely clear their system of the parasite, once infected, compared with California quail. If a large proportion of Gambel's quail are unable to completely flush *H. lophortyx* from their system, this may lead to a relatively high prevalence of low‐intensity, chronic infections. Because infection intensities are usually highest during the early stages of infection (e.g., Ahmed & Mohammed, 1978; Cepeda et al., 2019), the few individuals with relatively high infection intensities seen in California quail may simply reflect a handful of newly infected individuals.

Under this scenario, hybrid quail may have inherited resistance alleles from parental California quail. Chronic *Haemoproteus* infections have been demonstrated to have fitness costs in other host systems (Puente et al., 2010; Asghar et al., 2011; Asghar et al., 2015; but see Ortego et al., 2008). If chronic infections in Gambel's quail lead individuals to have lower fitness compared with hybrid quail, hybridization and backcrossing may facilitate the introgression of resistance alleles, and we would expect species barriers to be eroded.

We robustly characterized the prevalence of *H. lophortyx* infection and quantified the intensity of infection across California, Gambel's, and hybrid quail. However, more work is needed to tease apart the two mutually exclusive interpretations of our results presented above. Although past work has shown that *H. lophortyx* can have high fitness costs for quail, even resulting in death (Cardona et al., 2002; Mullens et al., 2006; O’Roke, 1930, 1932), we did not examine the relative fitness costs of this parasite for each host species, the differences between species in *H. lophortyx* tolerance, or the potential costs of chronic, low‐intensity infections. Given this, while we can infer the potential for *H. lophortyx* to influence species barrier dynamics, we cannot draw any definitive conclusions regarding the parasite‐driven maintenance or breakdown of species barriers, and future work should assess the relative fitness costs and course of infection of *H. lophortyx* across this hybrid zone. Furthermore, our study possesses several other limitations that future studies might work to address. For example, we scored individuals as either hybrids or parental species (Figure 1); however, because hybrids are not truly a single class, differences may exist between hybrids in parasite susceptibility or fitness costs, thereby affecting the influence of parasitism on gene flow (Derothe et al., 2004; Goldberg et al., 2005; Theodosopoulos et al., 2019). If most of the hybrids sampled in our study were highly backcrossed to California quail, this could explain the observed similarities between hybrid and California quail in prevalence and intensity of *H. lophortyx* infection. Furthermore, because interactions between different parasites may not be additive, coinfection dynamics can have drastic consequences for the effects of parasitism on gene flow, and studies which examine a single parasite may only capture a portion of the story (Bordes & Morand, 2011; Johnson & Hoverman, 2012; Rynkiewicz et al., 2015; Telfer et al., 2010; Theodosopoulos et al., 2019; Vaumourin et al., 2015). Lastly, we assumed that California quail, Gambel's quail, and their hybrids were all infected by a single lineage of *Haemoproteus* in this study. Although past work on *H. lophortyx* has relied on the morphological identification of this blood parasite (Atkinson & Van Riper, 1991b; Cardona et al., 2002; Herman & Glading, 1942; Mullens et al., 2006; O’Roke, 1928, 1930, 1932; Tarshis, 1955), a drawback of this approach is that different lineages may appear morphologically identical, while remaining genetically distinct (Bensch et al., 2000). This is especially relevant given the high host specificity of *Haemoproteus* lineages, and it is possible that different lineages may have disparate pathologies (Atkinson & Van Riper, 1991a; Ayadi et al., 2018; Bensch et al., 2000; Clark & Clegg, 2017; Loiseau et al., 2017).

Lastly, we found that patterns of infection varied across years; however, the cause(s) of these differences remains unclear. Increases in precipitation have been found to either increase host exposure rates (O'Connor et al., 2007, 2008; Bohrer et al., 2014; Shearer & Ezenwa, 2020) or decrease host susceptibility to parasitism (Ezenwa, 2004; Masi et al., 2012; Shearer & Ezenwa, 2020; Thurber et al., 2011) in other systems. Although El Niño Southern Oscillations caused precipitation to fluctuate across years in our study, it is unlikely that the differences in infection status and intensity seen between years were related to variation in precipitation (see Table S6 for a summary of how year relates to precipitation, infection status, and infection intensity.) Nevertheless, because hybridization has been shown to increase with increased precipitation (Gee, 2004), precipitation may work in tandem with *H. lophortyx* to mediate species barrier dynamics between California and Gambel's quail. Furthermore, it is possible that the proportion of infected individuals differed between years because of interannual variation in the synchronization of host phenology and vector phenology or in the temporal overlap of different vector life stages (MacDonald et al., 2020). For example, models by MacDonald et al. (2020) demonstrate that the basic reproductive number (i.e., the average number of new infections a single infected tick causes) of *Borrelia burgdorferi* (a causative agent of Lyme disease) is highest when the larval activity of its tick vector is clustered around the peak infection prevalence of its mouse host.

In summary, we found that infection prevalence was higher, while infection intensity was lower, in Gambel's quail, compared with hybrid and California quail, suggesting that *H. lophortyx* infection has the potential to influence species barrier dynamics in this system. Future work should focus on examining the fitness consequences and course of infection of *H. lophortyx*, as well as the diversity of *Haemoproteus* lineages and their distribution across this quail hybrid zone.

## CONFLICT OF INTEREST

None declared.

## AUTHOR CONTRIBUTIONS

**Allison M. Roth:** Conceptualization (equal); data curation (lead); formal analysis (lead); funding acquisition (supporting); investigation (equal); methodology (equal); project administration (equal); resources (equal); software (equal); supervision (equal); validation (equal); visualization (supporting); writing–original draft (lead); writing–review and editing (lead). **Carl N. Keiser:** Conceptualization (equal); data curation (supporting); formal analysis (supporting); funding acquisition (lead); investigation (equal); methodology (supporting); project administration (equal); resources (equal); software (equal); supervision (equal); validation (equal); visualization (lead); writing–original draft (supporting); writing–review and editing (equal). **Judson B. Williams:** Conceptualization (equal); data curation (supporting); formal analysis (supporting); funding acquisition (supporting); investigation (equal); methodology (equal); project administration (equal); resources (equal); software (equal); supervision (equal); validation (equal); visualization (supporting); writing–original draft (supporting); writing–review and editing (supporting). **Jennifer M. Gee:** Conceptualization (equal); data curation (supporting); formal analysis (supporting); funding acquisition (lead); investigation (equal); methodology (equal); project administration (equal); resources (equal); software (equal); supervision (equal); validation (equal); visualization (supporting); writing–original draft (supporting); writing–review and editing (supporting).

## Supporting information

Fig S1

Supplementary Material

## Data Availability

The data associated with this manuscript are available on Figshare (https://doi.org/10.6084/m9.figshare.12217958).
