# Supplementary figures and images for: Prevalence and intensity of avian malaria in a quail hybrid zone
Source: Ecol Evol. 2021 May 19;11(12):8123–35. doi: 10.1002/ece3.7645 (PMC8216944; doi:10.1002/ece3.7645)

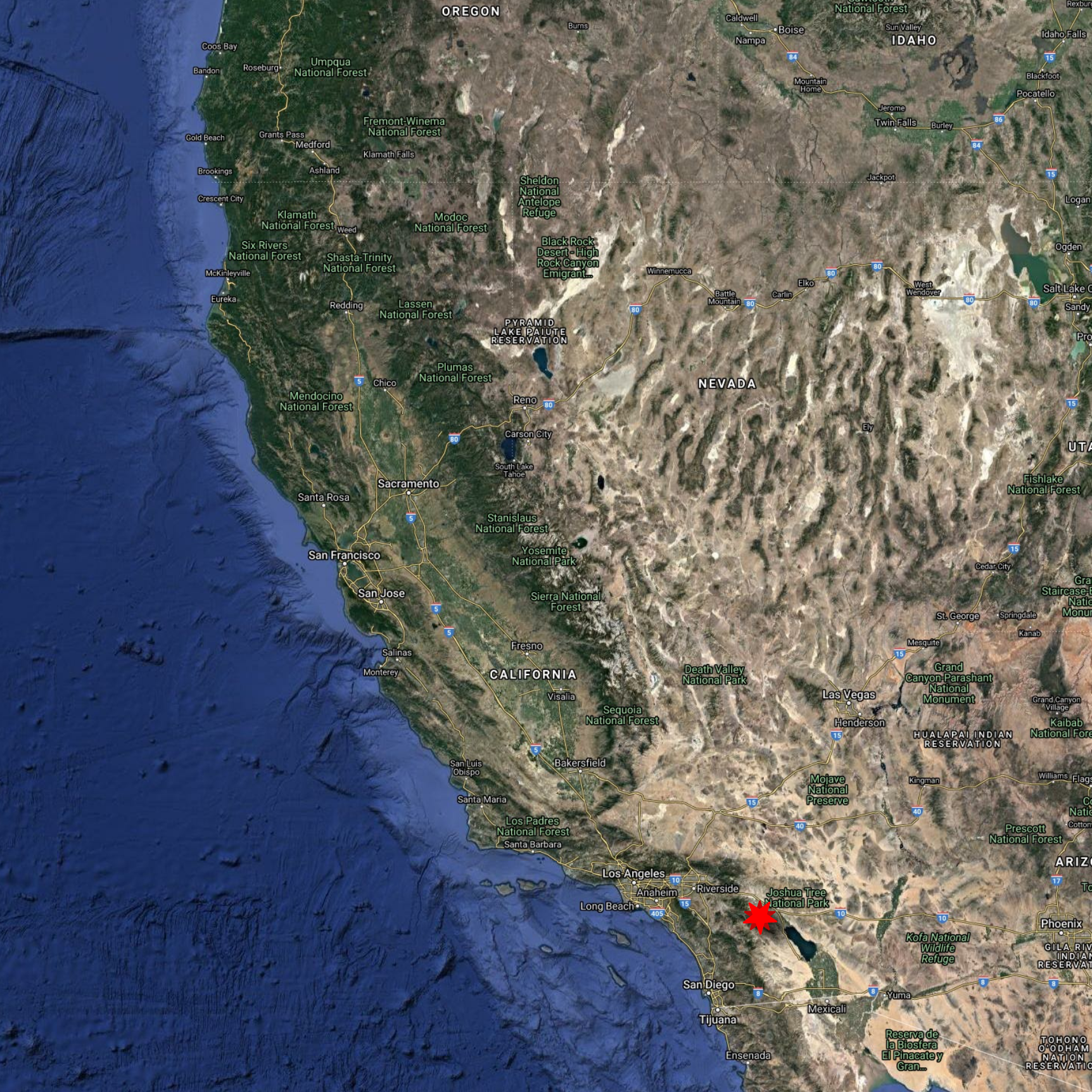

Supplement: Supplementary file 1 — Fig S1 [file ECE3-11-8123-s001.png]
