# Supplementary material for: Prevalence and intensity of avian malaria in a quail hybrid zone
Source: Ecol Evol. 2021 May 19;11(12):8123–35. doi: 10.1002/ece3.7645 (PMC8216944; doi:10.1002/ece3.7645)
Supplement: Supplementary file 2 — Supplementary Material [file ECE3-11-8123-s002.docx]

**APPENDIX 1**

When rerunning analyses using the subset of data where at least 10,000 erythrocytes were examined for each sample, our results were identical to the results from models where we scanned each blood smear for 15 minutes at 100x magnification, with one small exception (Tables S4 – S5 and S7– S8). In the model where we scanned blood smears for 15 minutes, Gambel’s and hybrid quail differed significantly in intensity of infection. In contrast, when we limited data to 10,000 or more erythrocytes, this difference was marginally nonsignificant (p = 0.050; Table S5). As in the models that used data where we scanned blood smears for 15 minutes, when at least 10,000 erythrocytes were examined, there was an overall effect of species on infection status (Chi-squared = 7.778, df = 2, p = 0.020) and intensity (Chi-squared = 7.361, df = 2, p = 0.025), as well as an overall effect of year on infection status (Chi-squared = 18.778, df = 3, p < 0.001) and intensity (Chi-squared = 17.330, df = 3, p = 0.001). Sex did not appear to have an overall effect on either infection status (Chi-squared = 0.045, df = 1, p = 0.832) or intensity (Chi-squared = 1.087, df = 1, p = 0.297).


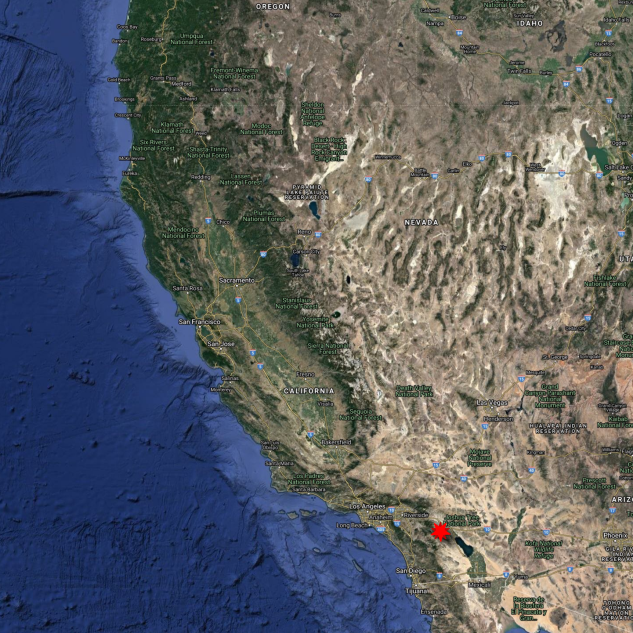


**Figure S1.** Map of the California coast made using QGIS (QGIS.org 2021). Royal Carrizo, our study site, is indicated by the red star.

**Table S1.** The number of samples obtained for each species in each year.

| **Predictor** | **California** | **Hybrid** | **Gambel’s** |
| --- | --- | --- | --- |
| 1998 | 25 | 23 | 13 |
| 1999 | 6 | 19 | 7 |
| 2000 | 43 | 51 | 8 |
| 2001 | 3 | 8 | 2 |

**Table S2.** Output of a generalized linear mixed model with a binomial error distribution showing the effects of species (i.e., California quail, Gambel’s quail, California × Gambel’s quail hybrid), year (1998, 1999, 2000, 2001), and sex on *Haemoproteus lophortyx* infection status, when infection was determined by scanning blood smears for 15 minutes at 100x magnification (N = 208). Individual identity and month were included as random effects.

| **Predictor** | **Est ± SE** | **Z** | **p** |  |
| --- | --- | --- | --- | --- |
| **Reference Class: 1998** |  |  |  |  |
| Species: California Quail | -1.394 ± 0.519 | -2.685 | **0.007** |  |
| Species: Hybrid Quail | -1.406 ± 0.504 | -2.788 | **0.005** |  |
| Year: 1999 | -2.734 ± 0.817 | -3.346 | **0.001** |  |
| Year: 2000 | -0.098 ± 0.374 | -0.261 | 0.794 |  |
| Year: 2001 | -0.972 ± 0.764 | -1.272 | 0.204 |  |
| Sex: Male | 0.173 ± 0.341 | 0.507 | 0.612 |  |
| **Reference Class: 1999** |  |  |  |  |
| Species: California Quail | -1.394 ± 0.519 | -2.685 | **0.007** |  |
| Species: Hybrid Quail | -1.406 ± 0.504 | -2.788 | **0.005** |  |
| Year: 1998 | 2.734 ± 0.817 | 3.346 | **0.001** |  |
| Year: 2000 | 2.637 ± 0.805 | 3.276 | **0.001** |  |
| Year: 2001 | 1.762 ± 1.050 | 1.679 | 0.093 |  |
| Sex: Male | 0.173 ± 0.341 | 0.507 | 0.612 |  |
| **Reference Class: 2000** |  |  |  |  |
| Species: California Quail | -1.394 ± 0.519 | -2.685 | **0.007** |  |
| Species: Hybrid Quail | -1.406 ± 0.504 | -2.788 | **0.005** |  |
| Year: 1998 | 0.098 ± 0.374 | 0.261 | 0.794 |  |
| Year: 1999 | -2.637 ± 0.805 | -3.276 | **0.001** |  |
| Year: 2001 | -0.874 ± 0.765 | -1.142 | 0.253 |  |
| Sex: Male | 0.173 ± 0.341 | 0.507 | 0.612 |  |
| **Reference Class: 2001** |  |  |  |  |
| Species: California Quail | -1.394 ± 0.519 | -2.685 | **0.007** |  |
| Species: Hybrid Quail | -1.406 ± 0.504 | -2.788 | **0.005** |  |
| Year: 1998 | 0.972 ± 0.764 | 1.272 | 0.204 |  |
| Year: 1999 | -1.762 ± 1.050 | -1.679 | 0.093 |  |
| Year: 2000 | 0.874 ± 0.765 | 1.142 | 0.253 |  |
| Sex: Male | 0.173 ± 0.341 | 0.507 | 0.612 |  |

The results presented in each subsection of this table represent the same model with different years coded as the reference class. Presenting the same model with each year coded as the reference class allows for a comparison of *H. lophortyx* infection between each pair of years. The reference class for species is Gambel’s quail, and the reference class for sex is female.

**Table S3.** Output of a generalized linear mixed model with a binomial error distribution showing the effects of species (i.e., California quail, Gambel’s quail, California × Gambel’s quail hybrid), year (1998, 1999, 2000, 2001), and sex on the intensity of *Haemoproteus lophortyx* infection (i.e., proportion of infected erythrocytes), when infection was determined by scanning blood smears for 15 minutes at 100x magnification (N = 69). Individual identity and month were included as random effects.

| **Predictor** | **Est ± SE** | **Z** | **p** |
| --- | --- | --- | --- |
| **Reference Class: 1998** |  |  |  |
| Species: California Quail | 1.416 ± 0.486 | 2.913 | **0.004** |
| Species: Hybrid Quail | 0.942 ± 0.469 | 2.010 | **0.044** |
| Year: 1999 | 0.319 ± 0.305 | 1.045 | 0.296 |
| Year: 2000 | -0.088 ± 0.290 | -0.304 | 0.761 |
| Year: 2001 | -0.770 ± 0.827 | -0.932 | 0.351 |
| Sex: Male | -0.244 ± 0.356 | -0.686 | 0.493 |
| **Reference Class: 1999** |  |  |  |
| Species: California Quail | 1.416 ± 0.486 | 2.913 | **0.004** |
| Species: Hybrid Quail | 0.942 ± 0.469 | 2.010 | **0.044** |
| Year: 1998 | -0.319 ± 0.305 | -1.045 | 0.296 |
| Year: 2000 | -0.407 ± 0.101 | -4.048 | **<0.001** |
| Year: 2001 | -1.089 ± 0.811 | -1.343 | 0.179 |
| Sex: Male | -0.244 ± 0.356 | -0.686 | 0.493 |
| **Reference Class: 2000** |  |  |  |
| Species: California Quail | 1.416 ± 0.486 | 2.913 | **0.004** |
| Species: Hybrid Quail | 0.942 ± 0.469 | 2.010 | **0.044** |
| Year: 1998 | 0.088 ± 0.290 | 0.304 | 0.761 |
| Year: 1999 | 0.407 ± 0.101 | 4.048 | **<0.001** |
| Year: 2001 | -0.682 ± 0.805 | -0.848 | 0.397 |
| Sex: Male | -0.244 ± 0.356 | -0.686 | 0.493 |
| **Reference Class: 2001** |  |  |  |
| Species: California Quail | 1.416 ± 0.486 | 2.913 | **0.004** |
| Species: Hybrid Quail | 0.942 ± 0.469 | 2.010 | **0.044** |
| Year: 1998 | 0.770 ± 0.827 | 0.932 | 0.351 |
| Year: 1999 | 1.089 ± 0.811 | 1.343 | 0.179 |
| Year: 2000 | 0.682 ± 0.805 | 0.848 | 0.397 |
| Sex: Male | -0.244 ± 0.356 | -0.686 | 0.493 |

The results presented in each subsection of this table represent the same model with different years coded as the reference class. Presenting the same model with each year coded as the reference class allows for a comparison of *H. lophortyx* infection intensity between each pair of years. The reference class for species is Gambel’s quail, and the reference class for sex is female.

**Table S4.** Output of a generalized linear mixed model with a binomial error distribution showing the effects of species (i.e., California quail, Gambel’s quail, California × Gambel’s quail hybrid), year (1998, 1999, 2000, 2001), and sex on *Haemoproteus lophortyx* infection status, when infection was determined by examining at least 10,000 erythrocytes (N = 189). Individual identity and month were included as random effects.

| **Predictor** | **Est ± SE** | **Z** | **p** |
| --- | --- | --- | --- |
| **Reference Class: California Quail** | |  |  |
| Species: Hybrid Quail | 0.009 ± 0.369 | 0.024 | 0.981 |
| Species: Gambel’s Quail | 1.370 ± 0.546 | 2.509 | **0.012** |
| Year: 1999 | -2.702 ± 0.824 | -3.278 | **0.001** |
| Year: 2000 | -0.272 ± 0.391 | -0.696 | 0.486 |
| Year: 2001 | -1.044 ± 0.767 | -1.362 | 0.173 |
| Sex: Male | 0.085 ± 0.356 | 0.239 | 0.811 |
| **Reference Class: Hybrid Quail** |  |  |  |
| Species: California Quail | -0.009 ± 0.369 | -0.024 | 0.981 |
| Species: Gambel’s Quail | 1.361 ± 0.523 | -2.602 | **0.009** |
| Year: 1999 | -2.702 ± 0.824 | -3.278 | **0.001** |
| Year: 2000 | -0.272 ± 0.391 | -0.696 | 0.486 |
| Year: 2001 | -1.044 ± 0.767 | -1.362 | 0.173 |
| Sex: Male | 0.085 ± 0.356 | 0.239 | 0.811 |
| **Reference Class: Gambel’s Quail** |  |  |  |
| Species: California Quail | -1.370 ± 0.546 | -2.509 | **0.012** |
| Species: Hybrid Quail | -1.361 ± 0.523 | -2.602 | **0.009** |
| Year: 1999 | -2.702 ± 0.824 | -3.278 | **0.001** |
| Year: 2000 | -0.272 ± 0.391 | -0.696 | 0.486 |
| Year: 2001 | -1.044 ± 0.767 | -1.362 | 0.173 |
| Sex: Male | 0.085 ± 0.356 | 0.239 | 0.811 |

The results presented in each subsection of this table represent the same model with different species coded as the reference class. Presenting the same model with each species coded as the reference class allows for a comparison of *H. lophortyx* infection status between each pair of species. For year, the reference class is 1998 (see Table S7 for a comparison between years). For sex, the reference class is female.

**Table S5.** Output of a generalized linear mixed model with a binomial error distribution showing the effects of species (i.e., California quail, Gambel’s quail, California × Gambel’s quail hybrid), year (1998, 1999, 2000, 2001), and sex on the intensity of *Haemoproteus lophortyx* infection (i.e., proportion of infected erythrocytes), when infection was determined by examining at least 10,000 erythrocytes (N = 63). Individual identity and month were included as random effects. Fixed effects that differ in significance between models using data where at least 10,000 erythrocytes were examined and models using data where at least 30 fields of view at 100x magnification were examined are shaded in grey.

| **Predictor** | **Est ± SE** | **Z** | **p** |
| --- | --- | --- | --- |
| **Reference Class: California Quail** | |  |  |
| Species: Hybrid Quail | -0.494 ± 0.407 | -1.216 | 0.224 |
| Species: Gambel’s Quail | -1.482 ± 0.537 | -2.762 | **0.006** |
| Year: 1999 | 0.370 ± 0.319 | 1.160 | 0.246 |
| Year: 2000 | -0.037 ± 0.304 | -0.123 | 0.903 |
| Year: 2001 | -0.753 ± 0.857 | -0.879 | 0.380 |
| Sex: Male | -0.399 ± 0.391 | -1.020 | 0.308 |
| **Reference Class: Hybrid Quail** |  |  |  |
| Species: California Quail | 0.494 ± 0.407 | 1.216 | 0.224 |
| Species: Gambel’s Quail | -0.988 ± 0.504 | -1.959 | 0.050 |
| Year: 1999 | 0.370 ± 0.319 | 1.160 | 0.246 |
| Year: 2000 | -0.037 ± 0.304 | -0.123 | 0.903 |
| Year: 2001 | -0.753 ± 0.857 | -0.879 | 0.380 |
| Sex: Male | -0.399 ± 0.391 | -1.020 | 0.308 |
| **Reference Class: Gambel’s Quail** |  |  |  |
| Species: California Quail | 1.482 ± 0.537 | 2.762 | **0.006** |
| Species: Hybrid Quail | 0.988 ± 0.504 | 1.959 | **0.050** |
| Year: 1999 | 0.370 ± 0.319 | 1.160 | 0.246 |
| Year: 2000 | -0.037 ± 0.304 | -0.123 | 0.903 |
| Year: 2001 | -0.753 ± 0.857 | -0.879 | 0.380 |
| Sex: Male | -0.399 ± 0.391 | -1.020 | 0.308 |

The results presented in each subsection of this table represent the same model with different species coded as the reference class. Presenting the same model with each species coded as the reference class allows for a comparison of *H. lophortyx* infection intensity between each pair of species. For year, the reference class is 1998 (see Table S8 for a comparison between years). For sex, the reference class is female.

**Table S6.** Summary of winter precipitation across our four study years and how infection prevalence and intensity differed across years, relative to one another. We found that significantly fewer individuals were infected in 1999 than in 1998 or 2000, and, once infected, individuals expressed higher infection intensities in 1999 compared to 2000. Because 1) there was a significant difference in the proportion of infected individuals between the first and second and between the first and forth driest years, but not between the first and third driest years, and because 2) the only significant difference in infection intensity occurred between 1999 and 2000, which were the two study years with the lowest levels of precipitation, it is unlikely that interannual variation in precipitation drove the differences in infection status and intensity seen between years in our study. Furthermore, given that we had a very limited sample size in 1999 and 2001, for the analysis examining the intensity of infection, both the results and any interpretations of the underlying mechanisms must be taken with caution.

| **Year** | **Precipitation (mm)** | **Infection Prevalence** | **Infection Intensity** |
| --- | --- | --- | --- |
| 1998 | 149.2 | High | - |
| 1999 | 28.3 | Low | High |
| 2000 | 59.3 | High | Low |
| 2001 | 81.1 | - | - |

We calculated winter precipitation from December 1 (of the preceding year) – February 28 for each year of our study using data acquired from the University of California, Boyd Deep Canyon Desert Research Center (<http://deepcanyon.ucnrs.org>). We examined winter precipitation rather than annual precipitation because 1) winter is when the bulk of precipitation occurs at our field site and 2) past work has demonstrated that winter precipitation strongly predicts annual breeding success and hybridization in quail (Gullion 1960; Francis 1970; Leopold 1977; Brown et al. 1998; Calkins et al. 1999; Gee 2004).

**Table S7.** Output of a generalized linear mixed model with a binomial error distribution showing the effects of species (i.e., California quail, Gambel’s quail, California × Gambel’s quail hybrid), year (1998, 1999, 2000, 2001), and sex on *Haemoproteus lophortyx* infection status, when infection was determined by examining at least 10,000 erythrocytes (N = 189). Individual identity and month were included as random effects.

| **Predictor** | **Est ± SE** | **Z** | **p** |
| --- | --- | --- | --- |
| **Reference Class: 1998** |  |  |  |
| Species: California Quail | -1.370 ± 0.546 | -2.509 | **0.012** |
| Species: Hybrid Quail | -1.361 ± 0.523 | -2.602 | **0.009** |
| Year: 1999 | -2.702 ± 0.824 | -3.278 | **0.001** |
| Year: 2000 | -0.272 ± 0.391 | -0.696 | 0.486 |
| Year: 2001 | -1.044 ± 0.767 | -1.362 | 0.173 |
| Sex: Male | 0.085 ± 0.356 | 0.239 | 0.811 |
| **Reference Class: 1999** |  |  |  |
| Species: California Quail | -1.370 ± 0.546 | -2.509 | **0.012** |
| Species: Hybrid Quail | -1.361 ± 0.523 | -2.602 | **0.009** |
| Year: 1998 | 2.702 ± 0.824 | 3.278 | **0.001** |
| Year: 2000 | 2.430 ± 0.807 | 3.012 | **0.003** |
| Year: 2001 | 1.658 ± 1.044 | 1.587 | 0.112 |
| Sex: Male | 0.085 ± 0.356 | 0.239 | 0.811 |
| **Reference Class: 2000** |  |  |  |
| Species: California Quail | -1.370 ± 0.546 | -2.509 | **0.012** |
| Species: Hybrid Quail | -1.361 ± 0.523 | -2.602 | **0.009** |
| Year: 1998 | 0.272 ± 0.391 | 0.696 | 0.486 |
| Year: 1999 | -2.430 ± 0.807 | -3.012 | **0.003** |
| Year: 2001 | -0.772 ± 0.755 | -1.023 | 0.306 |
| Sex: Male | 0.085 ± 0.356 | 0.239 | 0.811 |
| **Reference Class: 2001** |  |  |  |
| Species: California Quail | -1.370 ± 0.546 | -2.509 | **0.012** |
| Species: Hybrid Quail | -1.361 ± 0.523 | -2.602 | **0.009** |
| Year: 1998 | 1.044 ± 0.767 | 1.362 | 0.173 |
| Year: 1999 | -1.658 ± 1.044 | -1.587 | 0.112 |
| Year: 2000 | 0.772 ± 0.755 | 1.023 | 0.306 |
| Sex: Male | 0.085 ± 0.356 | 0.239 | 0.811 |

The results presented in each subsection of this table represent the same model with different years coded as the reference class. Presenting the same model with each year coded as the reference class allows for a comparison of *H. lophortyx* infection status between each pair of years. The reference class for species is Gambel’s quail, and the reference class for sex is female.

**Table S8.** Output of a generalized linear mixed model with a binomial error distribution showing the effects of species (i.e., California quail, Gambel’s quail, California × Gambel’s quail hybrid), year (1998, 1999, 2000, 2001), and sex on the intensity of *Haemoproteus lophortyx* infection (i.e., proportion of infected erythrocytes), when infection was determined by examining at least 10,000 erythrocytes (N = 63). Individual identity and month were included as random effects. Fixed effects that differ in significance between models using data where at least 10,000 erythrocytes were examined and models using data where at least 30 fields of view at 100x magnification were examined are shaded in grey.

| **Predictor** | **Est ± SE** | **Z** | **p** |
| --- | --- | --- | --- |
| **Reference Class: 1998** |  |  |  |
| Species: California Quail | 1.482 ± 0.537 | 2.762 | **0.006** |
| Species: Hybrid Quail | 0.988 ± 0.504 | 1.959 | 0.050 |
| Year: 1999 | 0.370 ± 0.319 | 1.160 | 0.246 |
| Year: 2000 | -0.037 ± 0.304 | -0.123 | 0.903 |
| Year: 2001 | -0.753 ± 0.857 | -0.879 | 0.380 |
| Sex: Male | -0.399 ± 0.391 | -1.020 | 0.308 |
| **Reference Class: 1999** |  |  |  |
| Species: California Quail | 1.482 ± 0.537 | 2.762 | **0.006** |
| Species: Hybrid Quail | 0.988 ± 0.504 | 1.959 | 0.050 |
| Year: 1998 | -0.370 ± 0.319 | -1.160 | 0.246 |
| Year: 2000 | -0.407 ± 0.101 | -4.050 | **<0.001** |
| Year: 2001 | -1.123 ± 0.837 | -1.342 | 0.180 |
| Sex: Male | -0.399 ± 0.391 | -1.020 | 0.308 |
| **Reference Class: 2000** |  |  |  |
| Species: California Quail | 1.482 ± 0.537 | 2.762 | **0.006** |
| Species: Hybrid Quail | 0.988 ± 0.504 | 1.959 | 0.050 |
| Year: 1998 | 0.037 ± 0.304 | 0.123 | 0.903 |
| Year: 1999 | 0.407 ± 0.101 | 4.050 | **<0.001** |
| Year: 2001 | -0.716 ± 0.831 | -0.862 | 0.389 |
| Sex: Male | -0.399 ± 0.391 | -1.020 | 0.308 |
| **Reference Class: 2001** |  |  |  |
| Species: California Quail | 1.482 ± 0.537 | 2.762 | **0.006** |
| Species: Hybrid Quail | 0.988 ± 0.504 | 1.959 | 0.050 |
| Year: 1998 | 0.753 ± 0.857 | 0.879 | 0.380 |
| Year: 1999 | 1.123 ± 0.837 | 1.342 | 0.180 |
| Year: 2000 | 0.716 ± 0.831 | 0.862 | 0.389 |
| Sex: Male | -0.399 ± 0.391 | -1.020 | 0.308 |

The results presented in each subsection of this table represent the same model with different years coded as the reference class. Presenting the same model with each year coded as the reference class allows for a comparison of *H. lophortyx* infection intensity between each pair of years. The reference class for species is Gambel’s quail, and the reference class for sex is female.

**Literature Cited**

Brown, D. E., Hagelin, J. C., Taylor, M. & Galloway, J. (1998). Gambel’s quail (*Callipepla gambelii*). Pp. 1–24 in A. Poole and F. Gill, eds. The birds of North America, no. 321 The Birds of North America, Inc., Philadelphia, PA; The American Ornithologists’ Union, Washington DC.

Calkins, J. D., J. C. Hagelin, and D. F. Lott. 1999. California quail (*Callipepla californica*). Pp. 1–32 in A. Poole and F. Gill, eds. The birds of North America. The Academy of Natural Sciences, Philadelphia, PA; The American Ornithologists’ Union, Washington DC.

Francis, W. J. (1970). The influence of weather on population fluctuations in California quail. J. Wildl. Manage., 34, 249-266.

Gee, J. M. (2004). Gene flow across a climatic barrier between hybridizing avian species, California and Gambel's quail (*Callipepla californica* and *C. gambelii*). Evolution, 58(5), 1108-1121.

Gullion, G. W. (1960). The ecology of Gambel's Quail in Nevada and the arid southwest. Ecology, 41(3), 518-536.

Leopold, A. S. (1977). The California Quail. University of California Press, Berkeley.

QGIS.org, 2021. QGIS Geographic Information System. QGIS Association. <http://www.qgis.org>.
